# An Efficient Two-Factor Authentication Scheme Based on the Merkle Tree

**DOI:** 10.3390/s20205735

**Published:** 2020-10-09

**Authors:** Xinming Yin, Junhui He, Yi Guo, Dezhi Han, Kuan-Ching Li, Arcangelo Castiglione

**Affiliations:** 1 Department of Computer Science and Engineering, East China University of Science and Technology, 130 Meilong Road, Shanghai 200237, China; yinxm6@163.com; 2 The Third Research Institute of Ministry of Public Security, 76 Yueyang Road, Shanghai 200031, China; 3 College of Information Engineering, Shanghai Maritime University, Shanghai 201306, China; hejunhui@stu.shmtu.edu.cn (J.H.); dzhan@shmtu.edu.cn (D.H.); 4 Department of Computer Science and Information Engineering (CSIE), Providence University, Taichung 43301, Taiwan; 5 School of Computer Science and Engineering, Anhui University of Science and Technology, Huainan 232001, China; 6 Department of Computer Science, University of Salerno, 84084 Fisciano, Italy; arcastiglione@unisa.it

**Keywords:** one-time password, two-factor authentication, hash chain, Merkle tree

## Abstract

The Time-based One-Time Password (TOTP) algorithm is commonly used for two-factor authentication. In this algorithm, a shared secret is used to derive a One-Time Password (OTP). However, in TOTP, the client and the server need to agree on a shared secret (i.e., a key). As a consequence, an adversary can construct an OTP through the compromised key if the server is hacked. To solve this problem, Kogan et al. proposed T/Key, an OTP algorithm based on a hash chain. However, the efficiency of OTP generation and verification is low in T/Key. In this article, we propose a novel and efficient Merkle tree-based One-Time Password (MOTP) algorithm to overcome such limitations. Compared to T/Key, this proposal reduces the number of hash operations to generate and verify the OTP, at the cost of small server storage and tolerable client storage. Experimental analysis and security evaluation show that MOTP can resist leakage attacks against the server and bring a tiny delay to two-factor authentication and verification time.

## 1. Introduction

Traditional authentication schemes based on usernames and passwords are affected by security vulnerabilities since they require users to provide usernames and passwords to prove their identity. Again, since a person chooses a password, such a password could be related to personal privacy and also affected by some regularity [1]. Therefore, an adversary can crack the password more efficiently, through consultations of and queries to a predefined password dictionary [2]. Furthermore, to remember a password quickly, most users use a fixed password for an extended period. On the other hand, with advances in the performance of processors, the possibility of the fixed password being cracked by brute force attacks also increases. In the end, to verify a user’s identity, the server needs to store the password, but can all servers guarantee the security of the password stored? Some servers still obey the bad practice of storing plaintext passwords. For example, six million plaintext passwords were exposed in the CSDN (China Software Developer Network) data breach, prompting users to change their passwords [3]. As is known, if a user uses the same password on multiple servers, whenever the password of one server is leaked, this password could be used for all the servers.

Consequently, multi-factor authentication has been widely applied to improve the security of traditional authentication schemes [4]. Besides fixed passwords, authentication factors typically include graphic passwords, biometrics, and One-Time Passwords (OTP). The two-factor authentication scheme based on OTP and fixed passwords is easy to implement, considering the cost. In particular, Google Authenticator [5] adopts the HMAC-based OTP (HOTP) [6] algorithm and the Time-based OTP (TOTP) [7] algorithm to implement a two-factor authentication scheme. However, HOTP and TOTP require that the client and the server share a secret key, bringing security risks when the server is compromised.

To deal with leakage attacks, Kogan et al. proposed T /Key [8]. T/Key is an OTP algorithm based on a hash chain. In this algorithm, the server stores the tail node of the hash chain for verification. Due to the pre-image resistance of the hash function, it is difficult for an adversary to forge the OTP from the information exposed by the server. Unfortunately, the efficiency of OTP generation and verification is low. Although Kogan et al. proposed a checkpoint method to increase the OTP generation efficiency at the cost of the client storage, the method still cannot improve the worst time complexity of verification.

SmartOTPs [9] is a solution that provides two-factor authentication for smart contract wallets. It uses a Merkle tree and hash chains to construct the OTP and uses the root of the Merkle tree stored in a smart contract to verify the OTP. Therefore, the leakage of smart contract information has little effect on the OTP security. Because SmartOTPs involves many entities and the scheme is complicated, it is not easily adopted by a simple client-server architecture.

The main goal of this paper is to propose an OTP algorithm that can resist leakage attacks. The proposed algorithm is efficient in terms of execution time and can be easily implemented. More precisely, inspired by T/Key and SmartOTPs, we propose an OTP algorithm based on the Merkle tree, called the Merkle tree-based One-Time Password (MOTP).

In detail, the main contributions are as follows:

1. We propose a novel OTP algorithm, referred to as the Merkle tree-based One-Time Password (MOTP). MOTP combines a Merkle tree with T/Key to construct many OTPs. Again, since it is affected by the limited number of generated OTPs, MOTP can be periodically reinitialized, reducing the probability for OTP to be cracked.
2. We improve the execution efficiency of the OTP algorithm. More precisely, MOTP improves the OTP verification performance of T/Key, at the cost of little server storage. In particular, since the time consumption of MOTP is milliseconds, it is difficult for users to notice such delays. On the other hand, compared with TOTP, the time consumption can increase the cost of brute force attacks for the adversary, and MOTP can resist leakage attacks against servers.
3. Security analysis and experiments prove the effectiveness of MOTP. More precisely, security analysis demonstrates that MOTP can resist leakage attacks against the server, and experimental results show that MOTP brings little delay to two-factor authentication.

The remainder of this article is organized as follows. In Section 2, we present the related works on OTP algorithms and two-factor authentication. Then, in Section 3, we propose the Merkle tree-based OTP (MOTP) algorithm and analyze the security of this algorithm. In Section 4, we describe the two-factor authentication scheme using QR codes to transfer OTPs generated by MOTP. In Section 5, we show the experimental evaluation and comparison. Finally, we present concluding remarks and future research directions in Section 6.

## 2. Related Work

The research on password security points out the contradiction between the security and usability of static passwords [10]. Authentication schemes based on Merkle trees and OTP have been proposed to improve the security of identity authentication.

Merkle trees have been applied in many authentication schemes. Li et al. [11] proposed a Merkle tree-based authentication to protect smart grids from message injection attacks and replay attacks. Huszti et al. [12] employed a Merkle tree to design an authentication and key exchange scheme for the cloud environment. SmartOTPs [9] applied a Merkle tree in the public blockchain to provide a two-factor authentication solution for smart contract wallets. In detail, SmartOTPs involves four entities: an authenticator (mobile), a client, a private key wallet, and a smart contract. Therefore, SmartOTPs is not quickly adopted by simple client-server architectures.

In terms of OTP, OTP algorithms are classified into two categories: one is based on a secret key; the other is based on a hash chain. Algorithms that are based on a secret key are HOTP [6] and TOTP [7]. These two algorithms require the client and the server to share a key and use the count value and timestamp to generate the OTP. However, when the server’s key gets exposed, the adversary can use it to forge the OTP.

Lamport first proposed an OTP algorithm based on a hash chain. Lamport’s OTP algorithm [13] uses a hash chain to construct the OTP. More precisely, the chain is generated by performing multiple times a one-way function (like a hash function) on a nonce. Each node value on the hash chain is regarded as an OTP. OTPs are consumed in order from the end to the head of the chain. Due to the pre-image resistance of the one-way function, it is difficult for an adversary to obtain a new OTP based on the consumed OTPs. However, the length of the hash chain is limited, and the algorithm can only provide a limited number of OTPs. Therefore, when the usage frequency of the OTPs is uncertain, it is difficult for the user to grasp the time when all OTPs ran out.

Bittl [14] used two hash functions to create a hash chain for infinite OTP generation. However, this algorithm requires that the client and the server share the same key, losing the advantages of Lamport’s OTP against leakage attacks. Park [15] used multiple short hash chains to defer construction and generate OTP infinitely. The feature of delayed construction reduces the initialization time of the algorithm, but Park’s algorithm increases the time cost of OTP generation and verification. Based on Lamport’s algorithm, Kogan et al. introduced a time gap to the OTP and proposed T/Key [8]. For example, in T/Key, if the validity time gap of each OTP is 30 seconds, a hash chain of $2^{20}$ length can be used for one year. Therefore, T/Key needs to periodically re-register the hash chain, reducing the possibility of the hash chain being cracked. Unfortunately, the main weakness of T/Key is that the OTP generation and verification execution efficiency are affected by the length of the hash chain. The longer the chain, the more hash operations are required for OTP generation and verification.

In terms of two-factor authentication, Erdem et al. proposed an OTP service, to make medium-sized enterprises adopt two-factor authentication efficiently [16]. Shirvanian et al. proposed LBD-QR-PIN [17], which is based on asymmetric encryption algorithms. More precisely, when verifying the user’s identity, the server uses the public key to encrypt a nonce and encodes it into a QR code. Then, the user scans the QR code using a mobile device, and such a device uses the private key to decrypt the nonce and then maps the nonce to a short-digit password through a hash function. It is difficult for an adversary to forge an OTP unless he/she obtains the private key stored on the user’s device. However, the performance of asymmetric encryption is poor.

## 3. Merkle Tree-Based One-Time Password Algorithm

To better understand the proposed algorithm, we first introduce the Merkle tree and explain why a Merkle tree can be used for OTP verification. Then, a naive Merkle tree-based one-time password scheme is proposed. Based on the naive scheme, we further improve the scheme, thereby improving execution efficiency, reducing network traffic, reducing client storage costs, and introducing a time gap to OTPs.

The notation used in the Merkle tree-based one-time password algorithm is shown in Table 1.

### 3.1. Merkle Tree

Merkle proposed the Merkle tree [18], which is widely used in blockchain to verify the integrity of data blocks [19,20,21].

Construction: The Merkle tree is a tree constructed bottom-up. More precisely, the tree discussed in this paper is a full binary tree. Assume that the height of the tree is $h_{m}$, and the tree owns $2^{h_{m}}$ data blocks $x_{i}$ and $y_{i}=hash\left(x_{i}\right),i\in \left[0,2^{h_{m}}-1\right]$, where $y_{i}$ is a leaf node value of the Merkle tree. Each value of the parent node is the hash of the concatenation of its children, $y_{parent}=hash\left(y_{left}|y_{right}\right)$, where | refers to concatenation.

**Figure 1:**
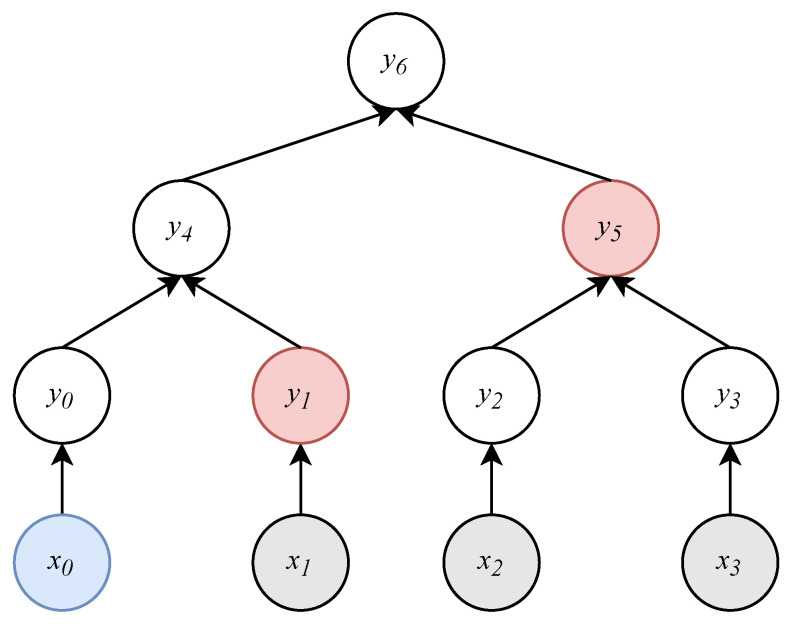
Merkle tree of height two. Each value of the parent node is the hash of the concatenation of its children, such as $y_{4}=hash\left(y_{0}|y_{1}\right)$.

In Figure 1, we show a Merkle tree of height two.

Proof path: Select a data block and find the sibling node of the corresponding leaf node. Based on this node, find the uncle node (the sibling node of its parent node) and repeat it until no uncle node can be found. The path composed of these nodes is called the proof path of the data block. For example, in Figure 1, the leaf node corresponding to the data block $x_{0}$ is $y_{0}$, and its sibling is $y_{1}$. Then, based on $y_{1}$, find its uncle node $y_{5}$, and $y_{5}$ has no uncle. Therefore, the proof path of $x_{0}$ is $\left\{y_{1},y_{5}\right\}$

Verification: Assume that a client has stored a whole Merkle tree and a server has the root node of the Merkle tree. The client sends a data block and its proof path to the server. The server uses the root node and the proof path to verify the data block. Take Figure 1 as an example. The server verifies $x_{0}$ by checking $hash\left(hash\left(hash\left(x_{0}\right)|y_{1}\right)|y_{5}\right)==y_{6}$. Actually, the server does not know $y_{1}$ is a right child of $y_{4}$, and $y_{5}$ is a right child of $y_{6}$, so the server cannot determine the concatenation order. We will handle this problem in Section 3.3.2. In general, a Merkle tree of height $h_{m}$ can verify $2^{h_{m}}$ data blocks.

### 3.2. A Naive Merkle Tree-Based One-Time Password Scheme

Using the Merkle tree to verify data blocks, we introduce a naive Merkle tree-based One-Time Password (MOTP) algorithm. This algorithm specifies data blocks as nonces and considers a nonce and its proof path as an OTP. Then, the server verification of the data block can be regarded as an OTP verification. The root node stored on the server can verify $2^{h_{m}}$ OTPs. Besides, due to the pre-image resistance of the hash function, it is difficult for adversaries to forge the OTP by using the root node leaked from the server.

### 3.3. An Improved Merkle Tree-Based One-Time Password Scheme

However, some problems arise in the naive scheme. We will discuss problems and solutions covering efficiency, network transmission, client storage, and validity.

#### 3.3.1. Efficiency

**Figure 2:**
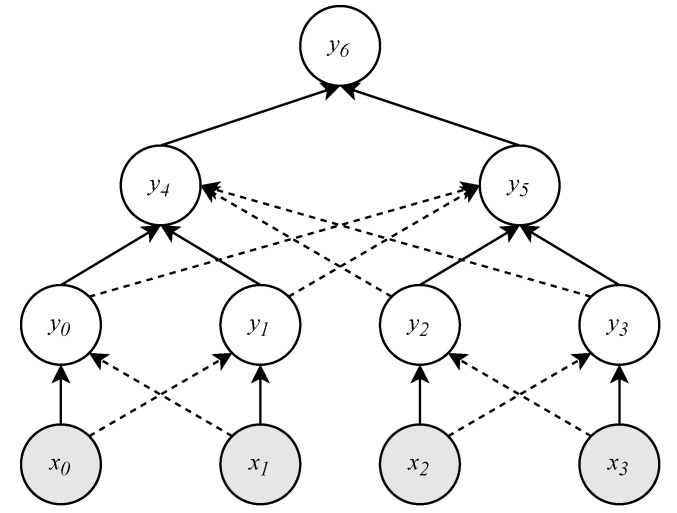
Associate each node with its uncle. The dotted line represents the new association. By taking $x_{0}$ as an example, we can get its proof path $\left\{y_{1},y_{5}\right\}$ quickly by the new association.

Assume that there is an association relationship between the sibling nodes. When obtaining a node’s uncle, we have to read its parent and then read the sibling of the parent. Therefore, to get a proof path of length *n*, we have to visit the nodes of a Merkle tree 2n times. This visit directly affects the generation efficiency of OTP. To improve efficiency, we associate each node with its uncle to reduce read times, as shown in Figure 2. Of course, the improvement of reading performance occurs at the expense of reference address memory.

#### 3.3.2. Network Transmission

As a part of the OTP, the proof path brings three problems.

The first is that the length of the proof path affects network transmission performance. In a Merkle tree of height $h_{m}$, the proof path includes $h_{m}$ nodes. Therefore, the higher the tree, the longer the proof path is. For example, when $h_{m}=10$ and the size of the hash value is 32B, the size of the proof path is 320 B.

The second is that some nodes in the proof paths frequently appear, which reduces the randomness of OTPs. Nodes with low depth appear more frequently in proof paths. For example, in Figure 1, we show four proof paths, namely {$y_{0},y_{5}$}, {$y_{1},y_{5}$}, {$y_{2},y_{4}$}, {$y_{3},y_{4}$}. The node depth of $y_{4}$ is one, and the frequency of $y_{4}$ in proof paths is 1/2.

**Figure 3:**
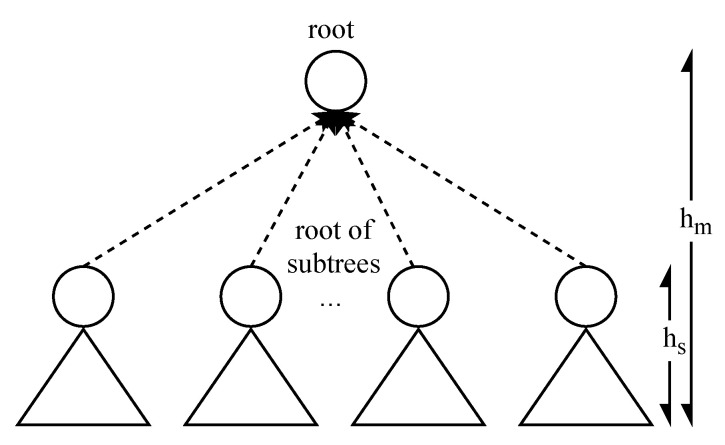
The tree of height $2^{h_{m}}$ is split into $2^{h_{m}-h_{s}}$ subtrees of height $2^{h_{s}}$.

To solve the problems mentioned above, we divide the Merkle tree into multiple subtrees, as shown in Figure 3. More precisely, the tree of height $h_{m}$ is divided into $2^{h_{m}-h_{s}}$ subtrees of height $h_{s}$. This operation shortens the proof path length to $h_{s}$. On the other hand, the server needs to store $2^{h_{m}-h_{s}}$ root nodes of subtrees for OTP verification. Hence, we need to find a tradeoff between server storage and transmission traffic. For example, when $h_{m}=10,h_{s}=7$, and the size of the hash value is 32 B, the original 1024 OTPs generate 352KB=1024×(10+1)×32B of traffic in the network transmission. Instead, after splitting the tree into subtrees, the traffic is reduced to 256KB=1024×(7+1)×32B. Conversely, the cost of server storage is increased from 32B to 256B. However, in terms of the saved traffic, the cost is worth it. Therefore, within the server’s storage capacity, the Merkle tree can be divided appropriately into multiple subtrees.

The third is that the server needs to know the position information of each node (left child or right child) in the proof path. For example, the client sends $x_{1}$, its proof path $\left\{y_{0},y_{5}\right\}$, and position information {0,1} (zero is left, and one is right) to the server. {0,1} means $y_{0}$ is a left child and $y_{5}$ is a right child. Then, the server knows $y_{0}$ is a left child and executes $hash(y_{0}|hash\left(x_{1}\right))$ instead of $hash(hash\left(x_{1}\right)|y_{0})$ to get $y_{4}$. $y_{5}$ is a right child, then the server executes $hash(y_{4}|y_{5})$ to get $y_{6}$.

To avoid the extra traffic caused by position information, we use *i* (the index of *x*) to replace position information. Assume the server receives $x_{i}$, its proof path, and *i*. If we know the index of a node, we can know the index of its parent by $i_{parent}=i_{child}/2+2^{h_{m}}$. If $i_{parent}$ is even, then the index of its uncle is $i_{uncle}=i_{parent}+1$. Otherwise, $i_{uncle}=i_{parent}-1$. Hence, the server can obtain the index of each node in the proof path from *i*. According to whether the index is even, the server can know whether the node is a left or right child.

Take an example: the server receives $x_{1}$, $\left\{y_{0},y_{5}\right\}$, and one. As we know, $x_{i}$ is associated with $y_{i}$, so $i_{parent}$ of $x_{1}$ is one, and $i_{uncle}$ is zero. Then, the server calculates $i_{parent}$ of $y_{0}$ and obtains four ($h_{m}=2$), so the $i_{uncle}$ of $y_{0}$ is five. The server gets a set of uncle indices {0,5} and obtains the position information {0,1}. In Section 3.3.4, the transmission traffic of *i* will be avoided.

#### 3.3.3. Client Storage

The number of OTPs provided by the Merkle tree increases exponentially with the tree height. Again, the higher the tree, the more storage the client requires, as shown in Table 2. The tree of height 10 owns $2^{10}$ nonces and $2^{11}-1$ nodes. When the size of the hash value and the nonce is 32 B, the tree needs 96 KB of storage.

**Figure 4:**
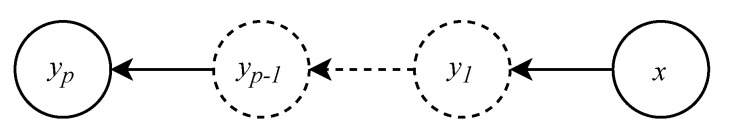
Space-time tradeoff of the hash chain. The nonce *x* in the figure is hashed *p* times to generate a hash chain, where $y_{i}=hash^{i}\left(x\right)$.

In this paper, we use the idea of “space-time tradeoff” [22] to decrease the storage space with increased execution time. More precisely, we use Lamport’s OTP algorithm to hash a nonce *p* times, to generate a hash chain of length p+1. The tail node of the hash chain serves as the leaf node of the Merkle tree, as shown in Figure 4. The client only stores the nonce *x* and the tail node $y_{p}$, and thus, the intermediate nodes of the hash chain must be re-calculated by using *x* when needed. Therefore, the storage cost of a node plus the appropriate execution cost can be exchanged for the storage cost of *p* nodes.

In addition to the leaf node, the hash chain can provide *p* OTPs. To construct a Merkle tree of height $h_{m}$, we need to construct $2^{h_{m}}$ hash chains of length p+1. The Merkle tree based on the hash chain can provide $2^{h_{m}}\;p$ OTPs. The client only needs to store the nonces used for the generation of hash chains and the whole Merkle tree constructed with leaf nodes. We call the node value on the hash chain otp (except for the leaf node) to avoid confusion. In MOTP, the OTP consists of otp and the corresponding proof path.

We remark that the above method increases the computing cost of the client and the server, but reduces the client’s storage burden. The Merkle tree of height 10 and the hash chain of length 1024 can provide 1,048,576 OTPs, while the client only requires 96 KB of storage. Without the hash chain, the client needs 96 MB to provide the same amount of OTP, as shown in Table 2. As a result, the solution with the hash chain saves about *p* times client storage than the solution without the hash chain.

#### 3.3.4. Validity

Since OTPs of MOTP are determined in advance, the more OTPs, the higher the probability of adversaries hitting an OTP by guessing attacks is. Furthermore, replay attacks and phishing attacks threaten the security of OTPs [23,24]. Therefore, we have to consider the validity of OTPs. Inspired by T/Key, when generating a hash chain, we make each OTP valid within a time gap of $t_{gap}$. The construction time of the Merkle tree is recorded as $t_{gen}$, which is shared by the client and the server.

**Figure 5:**
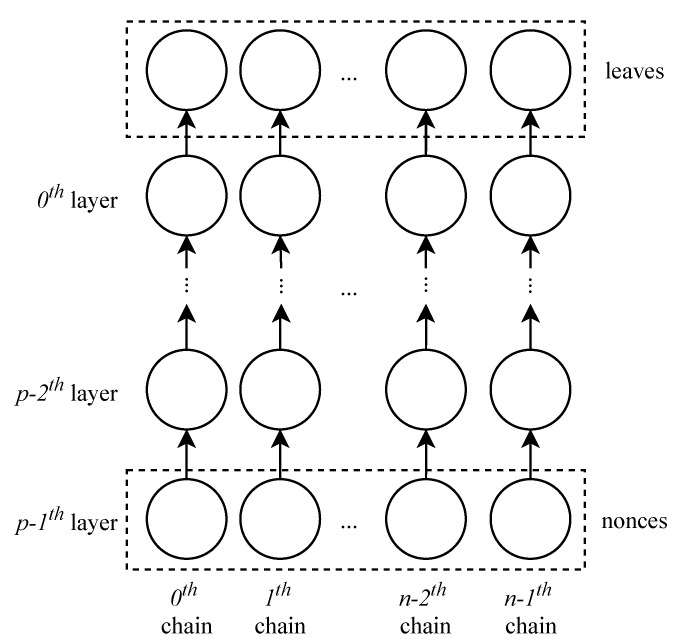
Hash chain layer.

After introducing $t_{gap}$ and $t_{gen}$, the client and the server can locate OTP by the device time. Nodes in the same index of the hash chain are recorded as the same layer. Since the tail node of the hash chain is a leaf node, the penultimate node is marked as the zeroth layer, as shown in Figure 5. OTP${}_{\left(i,j\right)}$ is an OTP of the $i^{th}$ layer of the $j^{th}$ hash chain. The calculation formula of *i* and *j* is as follows: % represents the modulo operation, and $t_{minus}$ represents the difference between the current time of the device and $t_{gen}$.

(1) $$\begin{array}{cc} i= & \left\lfloor t_{minus}/\left(2^{h_{m}}\cdot t_{gap}\right)\right\rfloor ,\\ j= & \left\lfloor \left(t_{minus}\%\left(2^{h_{m}}\cdot t_{gap}\right)\right)/t_{gap}\right\rfloor . \end{array}$$

The number of nodes in a layer of the hash chain is equal to the number of leaf nodes, that is $2^{h_{m}}$. $2^{h_{m}}\cdot t_{gap}$ shows the validity period of a layer of the hash chain. According to $t_{minus}$, we can know the layer *i* of the valid otp. Similarly, we can know the sequence number *j* of the hash chain of the valid otp. After the client gets (i,j), it hashes the $j^{th}$ nonce p−i−1 times to generate otp. The OTP is then composed of otp and proof corresponding to the $j^{th}$ nonce is sent to the server. After receiving the OTP, the server hashes otp *i* times and uses the root node of the ${\left\lfloor j/2^{h_{s}}\right\rfloor }^{th}$ subtree to verify the OTP. Hence, an expired OTP cannot pass the verification of the server. We remark that there is a threat of replay attacks in a time gap. The adversary can replay the same OTP in the time gap. Hence, the server should save the recently verified (i,j). When the server receives the OTP that has not expired, it checks whether the OTP’s (i,j) is equal to the saved (i,j). We remark that the server knows *i* by $t_{gap}$, so the transmission traffic of *i* mentioned in Section 3.3.2 can be avoided, which prevents the adversary from obtaining *i* in the transmission.

The validity period brings an implicit constraint; that is, the client and the server’s clocks must be synchronized. The problem can be solved using a time server to synchronize the client and the server clocks. Besides, the delay caused by algorithm execution and data transmission might also lead to failed verification. The solution to the problem can take a cue from TOTP, where the server uses multiple time gaps for OTP verification.

Since the number of nodes constructed by MOTP is limited and the validity period of each OTP is determined, MOTP can only work for a specified period. For example, assuming that $h_{m}=10$, p=1024, and $t_{gap}$ = 30 s, MOTP can be used for one year. Although the periodic initialization of MOTP will bring inconvenience to users, users can reinitialize the algorithm regularly. This operation can reduce the possibility of the root nodes stored on the server being cracked.

#### 3.3.5. Overall Schema

**Figure 6:**
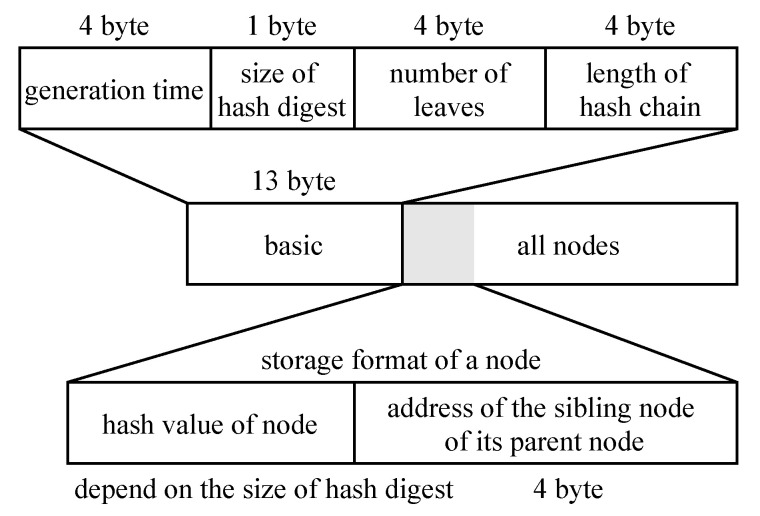
Binary format of the Merkle tree.

After the above optimizations, the binary format of the Merkle tree stored on the client is shown in Figure 6. The binary stream of the Merkle tree contains basic information and node information (including nonces).

The size of the hash digest defines the size of the hash value of each node and nonce. If the size is equal to four, the hash value requires four bytes of storage. The node information contains nonces and all nodes from the leaves to the roots of the subtrees. Suppose that $h_{m}=10$, $h_{s}=7$, and the size of hash is 32; the binary file storage of the Merkle tree is $110,317=13+\left(2^{10}+\left(2^{8}-1\right)\times 2^{10-7}\right)\times \left(32+4\right)$ bytes, i.e., about 108KB. The basic information needs 13 bytes. Each node needs 32 bytes for hash and four bytes for the address. The number of nodes is $2^{10}+\left(2^{8}-1\right)\times 2^{10-7}$, where $2^{10}$ is the number of nonces, $2^{8}-1$ is the number of nodes in a subtree, and $2^{10-7}$ is the number of subtrees.

**Figure 7:**
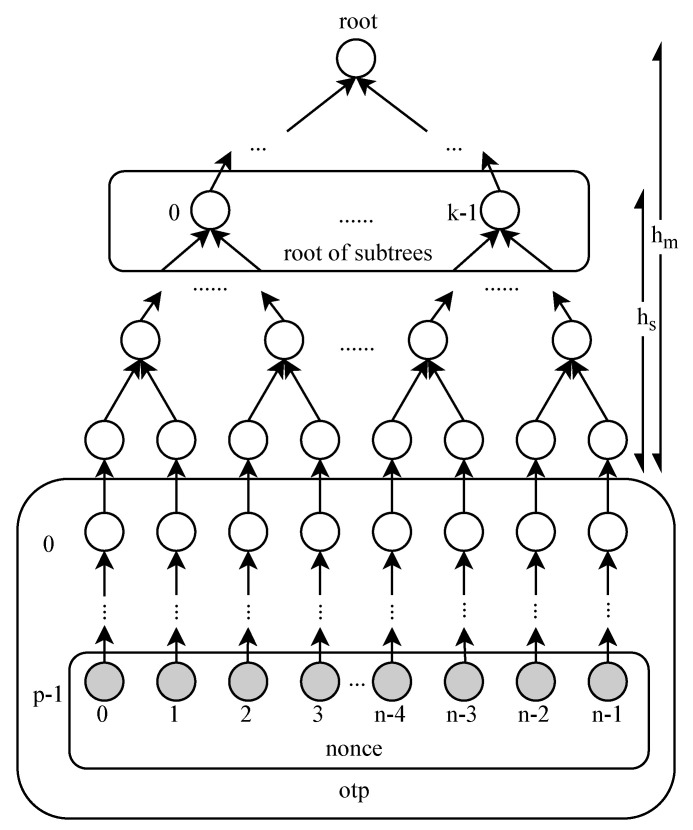
Merkle tree-based One-Time Password (MOTP) data structure.MOTP data structure

The MOTP data structure is shown in Figure 7. In detail, as can be seen from this figure, based on *n* nonces, we use *n* hash chains of length p+1, where $n=2^{h_{m}}$. The tail node of the hash chain is used as a leaf node, and then, a Merkle tree of height $h_{m}$ is constructed. The Merkle tree is split into *k* subtrees of height $h_{s}$, where $k=2^{h_{m}-h_{s}}$.

**Figure 8:**
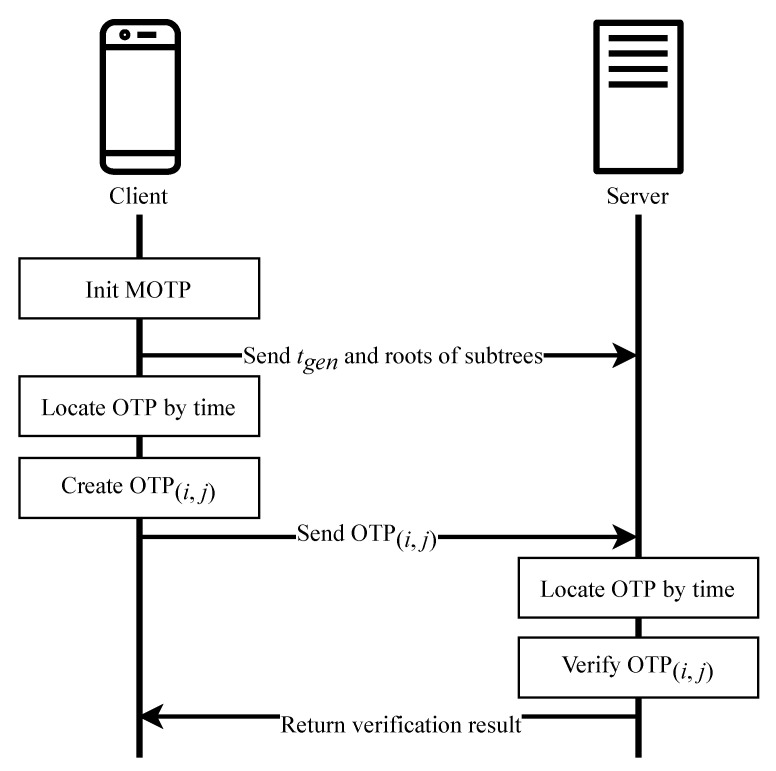
Execution process of MOTP.

The execution process of the MOTP is shown in Figure 8. The overall functioning of this algorithm includes the following steps:

Initialization: The client initializes the parameters and sends the initial value to the server, as described in Algorithm 1.

We remark that the MOTP algorithm parameters ($h_{m}$, $h_{s}$, *p*, $t_{gap}$, and the hash used algorithm) need to be negotiated by the client and the server in advance. Moreover, the communication between the client and the server is protected by TLS.

1. The client generates $2^{h_{m}}$ nonces and then performs *p* hash operations on each nonce to generate each hash chain.
2. The tail nodes of all hash chains serve as the leaf nodes and then generate a Merkle tree of height $2^{h_{m}}$.
3. Split the Merkle tree into $2^{h_{m}-h_{s}}$ subtrees of height $2^{h_{s}}$.
4. The client stores subtrees, the nonces, and $t_{gen}$, then it sends the root nodes of subtrees and $t_{gen}$ to the server through a secure channel.

**Algorithm 1:** MOTP initialization.

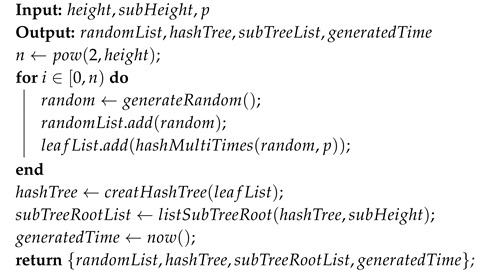



OTP generation: The client is responsible for the generation of the OTP, as described in Algorithm 2. More precisely, the client:

1. Calculates $t_{minus}$ and obtains (i,j), according to Equation (1).
2. Reads the $j^{th}$ nonce, $x_{j}$, and hashes it p−i−1 times to get otp.
3. Reads the proof path proof of $x_{j}$.
4. Sends {otp,proof} as $OTP_{\left(i,j\right)}$ to the server through a secure channel.

**Algorithm 2:** MOTP generation.**Input**: hashTree,subHeight,randomList,generatedTime **Output**: OTP timeMinus←now()−generatedTime; i,j←getPos(timeMinus); random←getRandom(j,randomList); otp←hashMultiTimes(random,p−i−1); proof←getProof(j,hashTree,subHeight); **return**{otp+proof};


OTP verification: The server is responsible for the verification of the OTP, as described in Algorithm 3. More precisely, the server:

1. Receives $OTP_{\left(i,j\right)}$ and parses it to get otp and proof.
2. Calculates $t_{minus}$ and gets (i,j) according to Equation (1).
3. Hashes otp*i* times to get $hash^{p-1}\left(x_{j}\right)$.
4. Calculates $\left\lfloor j/2^{h_{s}}\right\rfloor$, which is the sequence number of the subtree corresponding to $OTP_{\left(i,j\right)}$.
5. Uses proof and the root node value of the subtree to verify the correctness of $hash^{p-1}\left(x_{j}\right)$.

**Algorithm 3:** MOTP verification.**Input**: OTP,subHeight,subTreeRootList,generatedTime **Output**: verification result (true / false) timeMinus←now()−generatedTime; otp,proof←parse(OTP); i,j←getPos(timeMinus); data=hashMultiTimes(otp,i); subRoot←getSubRoot(j,subTreeRootList,subHeight); **return**verifyProof(data,proof,subRoot);


### 3.4. Security Analysis

Before going into the details of the security analysis, we first introduce the adversary model. Because MOTP and T/Key both are used in two-factor authentication, we refer to the adversary model of T/Key [8].

- The adversary can access all information stored on the server by using leakage attacks, but he/she cannot modify any value. Such information could be the root nodes.
- The adversary can perform at most *T* hash function computations until the server detects the attack and resets the Merkle tree.
- There is no malware on the client, and the adversary cannot get the OTP from the client. If the adversary can directly access the user’s device, he/she can carry out session hijacking, and authentication cannot prevent it. Then, the communication between the client and the server is protected by TLS, so the adversary cannot intercept the OTP through a man-in-the-middle attack.
- We divide adversaries into two types. Adversary $A_{1}$ performs guessing attacks to guess the correct OTP. Adversary $A_{2}$ intends to restore the Merkle tree by the root stored on the server and obtains all OTPs.

We define a uniform distribution hash function: [N]→[N], where $N=2^{n}$ and [N] is the set of $\left\{0,1,\ldots ,2^{n}-1\right\}$.

**Theorem** **1.** *Assume MOTP has the tree height $h_{m}$. $A_{1}$ obtains the root and the device’s current time from the server. Then, the adversary calculates the q, which is the number of hash operations required to convert to the leaf. If $A_{1}$ guesses the correct OTP, $A_{1}$ wins. Then,*

$$\mathrm{Pr}\left[A_{1}\;wins\right]\leq \frac{T}{(q+h_{m})N}$$

**Proof.**  The OTP consists of otp and a proof path of length $h_{m}$. To know whether the guessed OTP can pass the root’s verification, the adversary needs to perform $q+h_{m}$ hash operations. The root value is selected from [N]. Hence, the adversary can obtain an OTP, which can pass the verification with a probability of at most $\frac{T}{(q+h_{m})N}$. □

We remark that when an OTP is correctly guessed, there is also the possibility that such an OTP has expired.

**Theorem** **2.** *Assume the MOTP has tree height $h_{m}$ and hash chain length p. Let $A_{2}$ obtain the root stored on the server. If $A_{2}$ restores the Merkle tree from the root and obtains all OTPs, $A_{2}$ wins. Then,*

$$\mathrm{Pr}\left[A_{2}\;wins\right]\leq \frac{T}{(2^{h_{m}}-1)N}{\left(\frac{T}{pN}\right)}^{2^{h_{m}}}$$

**Proof.**  The tree of height $h_{m}$ has $2^{h_{m}}-1$ internal nodes and $2^{h_{m}}$ leaf nodes. Therefore, to generate a Merkle tree from the leaf nodes, the adversary has to perform $2^{h_{m}}-1$ hash operations and has at most $\frac{T}{2^{h_{m}}-1}$ chances to restore the Merkle tree. Since the range of the root is [0,N), the adversary has a probability of at most $\frac{T}{\left(2^{h_{m}}-1\right)N}$ to obtain the correct Merkle tree.

Assume that the adversary restores the tree and wants to gain all OTPs from the tree. The adversary selects a nonce, hashes it *p* times, and checks whether the result is equal to one of $2^{h_{m}}$ leaves. If they are equal, the adversary obtains a correct hash chain to generate OTPs. There are $2^{h_{m}}$ leaves, and the range of a leaf is [N], so the adversary obtains all correct hash chains with a probability of at most $\prod_{i=0}^{2^{h_{m}}-1}\frac{\frac{T}{p}-i}{N-i}$. Assume that $\frac{T}{p}<N$,

$$\prod_{i=0}^{2^{h_{m}}-1}\frac{\frac{T}{p}-i}{N-i}\leq {\left(\frac{T}{pN}\right)}^{2^{h_{m}}}$$

Hence, the adversary restores the tree and obtains all OTPs with a probability of at most:

$$\begin{array}{cc} \frac{T}{(2^{h_{m}}-1)N}\prod_{i=0}^{2^{h_{m}}-1}\frac{\frac{T}{p}-i}{N-i} & \leq \frac{T}{(2^{h_{m}}-1)N}{\left(\frac{T}{pN}\right)}^{2^{h_{m}}} \end{array}$$

 □

Through the above theorems and proofs, we show that even if the server is compromised, it is difficult for adversaries to guess a legal OTP or to obtain all OTPs.

## 4. Two-Factor Authentication

**Figure 9:**
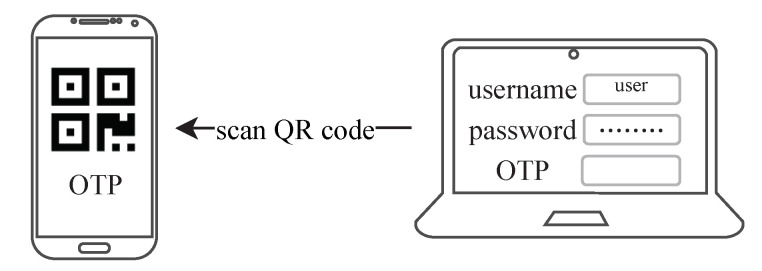
The application scans the QR code containing the OTP and obtains the OTP.

The two-factor authentication introduced in this paper is an authentication scheme composed of the static password and OTP. The two-factor authentication scheme based on OTP usually uses a mobile device as a client for generating OTPs. The verification of OTPs is performed by an application that requires identity authentication. In MOTP, the initialization and the generation of the OTP need to be done on the same side, so the mobile device must implement these two functions. Moreover, the long OTP provided by MOTP is not convenient for users to manually input. Although TOTP provides a truncation algorithm that converts the OTP to short digits, MOTP’s OTP contains a proof path that cannot be truncated directly. Therefore, we use QR codes [25] to transmit the OTP. In the following, we introduce two methods for using QR codes to achieve two-factor authentication. In the first method, the mobile device provides QR codes with MOTP’s initial information or the OTP. First, the user logs into the application through traditional username-password authentication and enables two-factor authentication. At this point, the application requires the user to provide MOTP’s initial information (root nodes and the generation time of the tree). The user then uses the mobile device to initialize MOTP and get a QR code encoded with initial information. Next, the application scans the QR code through the PC’s built-in camera and stores the initial information on the server. The next time the user logs into the application, an OTP is required besides providing the username and the password. Similarly, the application obtains an OTP through the QR code provided by the mobile, as shown in Figure 9. This method requires that the application can use a camera.

**Figure 10:**
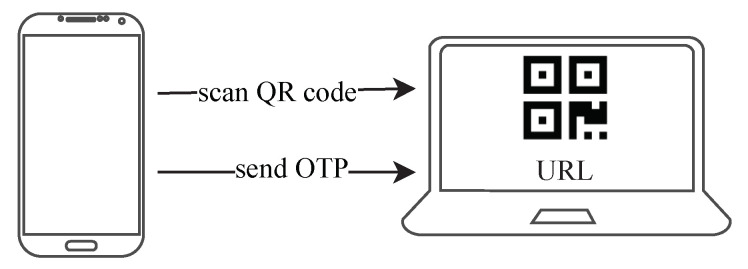
The mobile device scans the QR code containing a URL and sends the OTP to the URL.

In the latter method, the application provides QR codes with a URL. Due to the limitations of the previous method, we change the device that has to scan the QR code. Because most mobile devices provide a camera, we let the mobile device scan the QR codes, and the application provides the QR codes. After the mobile device scans the QR code containing a URL, it sends the initialization information or the OTP to the URL to replace the user’s manual input, as shown in Figure 10. Again, to prevent the mobile device from sending sensitive information to a malicious server, the user needs to confirm the URL’s domain.

## 5. Experimental Results and Discussion

To assess the MOTP algorithm’s performance, we designed and developed a proof of concept of this algorithm. We then tested the execution cost of the MOTP core functions and the transmission cost of OTP generated by MOTP. Finally, we compared MOTP with other schemes, especially T/Key and SmartOTPs.

### 5.1. Implementation

We implemented MOTP initialization and the OTP generation function using Android to evaluate the performance of MOTP on mobile devices. We also implemented MOTP initialization, OTP generation, and verification using Java to assess the performance of MOTP on PC devices.

The parameter configuration of the implemented MOTP is shown in Table 3. $h_{m}$ and *p* were set to 10 and 1024, respectively, which ensures enough OTPs generated by MOTP; $h_{s}$ was set to seven as a balance between server storage and transmission traffic, and $t_{gap}$ was set to 30 s to balance security and availability [7].

Based on the hash chain, the OTP algorithm usually stores the verified $OTP_{i}$ temporarily on the server. Then, when the server receives the new $OTP_{j}$, the server only needs to consider the hash operation from $OTP_{j}$ to $OTP_{i}$, which avoids the operation from $OTP_{i}$ to the tail node, thereby improving the verification efficiency. However, the verification efficiency of the current OTP will be affected by whether the previous OTP has been verified. It is difficult to show the verification efficiency of the worst case in the experiment. Therefore, the implemented OTP algorithms in the experiment will not adopt this approach.

### 5.2. Experiment

In the experiment, we mainly tested the execution cost of MOTP’s core functions. Considering that the OTP generated by MOTP is too long, we also needed to check the transmission cost of OTP.

#### 5.2.1. Execution Cost

We evaluated the time cost of the three core functions of the MOTP algorithm: initialization, OTP generation, and OTP verification. Initialization and OTP generation were tested on a mobile device equipped with a Qualcomm Snapdragon 855 Plus processor, 8GB memory, and Android 10 system, and a PC equipped with AMD 4-core A10-7400P processor, 12GB memory, and Ubuntu 19.10 system. OTP verification was only tested on the PC. Each core function was run more than 1000 times, and their average time consumption was counted. To demonstrate the execution efficiency of MOTP, we also tested T/Key.

Assuming that the other parameters are fixed, the value of the parameter *p* directly affects the validity period of T/Key and MOTP. For example, when T/Key’s *p* is $2^{20}$, $2^{22}$, $2^{24}$, and MOTP’s is $2^{10}$, $2^{12}$, $2^{14}$, the two schemes can provide OTPs for 1 year, 2 years, and 4 years, respectively.

Table 4 shows that the initialization time of the two schemes in the mobile environment is about five times slower than the PC. In the same device, the initialization time of MOTP is similar to T/Key. The length of the hash chain initialized in T/Key is $2^{10}$ times MOTP, and the number of hash chains initialized in MOTP is $2^{10}$ times T/Key. Therefore, the number of hash operations required for the initialization of the two schemes is equivalent. Although the initialization time cost of the two schemes is relatively high, waiting for five seconds in the mobile environment, the schemes can provide OTP for one year. Therefore, the time cost of initialization is worthwhile.

In T/Key and MOTP, the current time of the device determines which OTP is generated. More specifically, the number of hash operations required for the OTP generation and verification is affected by the device time. Furthermore, considering availability and security, we recommend using T/Key and MOTP with one-year validity. Hence, we modified the device time to test the time cost of OTP generation and verification in 12 months.

**Figure 11:**
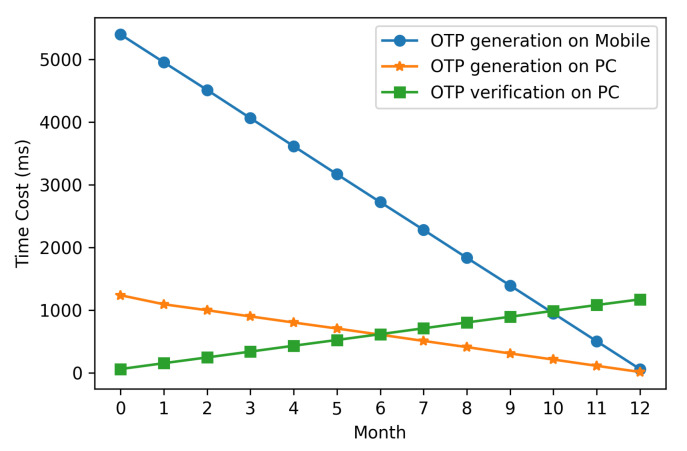
OTP generation and verification time cost of T/Key (valid for one year).

**Figure 12:**
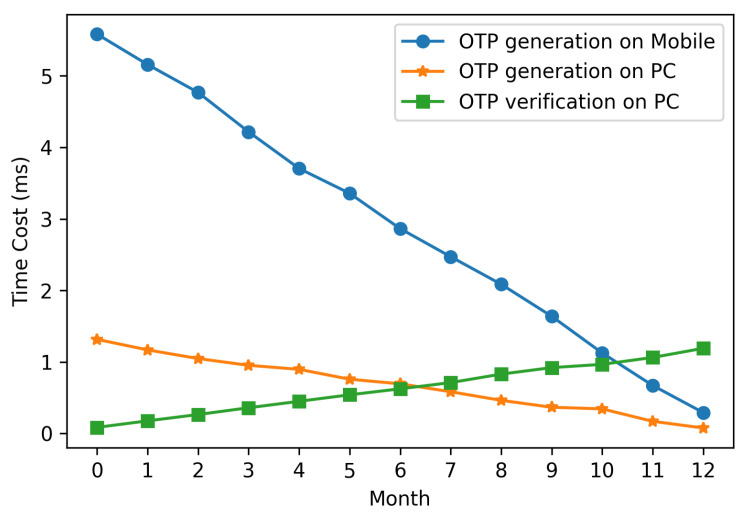
OTP generation and verification time cost of MOTP (valid for one year).

Figure 11 and Figure 12 show that MOTP’s time cost of OTP generation and verification is less than T/Key’s, and the time cost of MOTP is negligible. More specifically, the OTP generation and verification time cost of MOTP is one-thousandth that of T/Key. Because the OTP of the MOTP comes from $2^{10}$ hash chains of length $2^{10}$ and the OTP of T/Key comes from a hash chain of length $2^{20}$, the generation and verification time cost of MOTP is much lower than that of T/Key. Besides, we can find that the generation time cost decreases with the month, and the verification time increases with the month.

Given that, the total number of hash operations performed by the generation and verification is equal to the length of the hash chain. When the month is small, more hash operations are required for generation, and fewer operations are required for verification. Even in the worst case, MOTP’s OTP generation on the mobile device is below 6 ms, and verification on the PC is below 2 ms. Hence, the worst time of OTP generation and verification is 8 ms.

Therefore, the time cost is small, and the frequency of two-factor authentication is low (only used when the user logs in), so it is difficult for the user to perceive the delay. Finally, the time cost can burden adversaries and increase the cost of brute force cracking attacks.

#### 5.2.2. Transmission Cost

Considering the OTP length of MOTP, we need to test the transmission cost of the OTP. We use the client to issue 100,000 serial requests to the server automatically.

We only count the average time from issuing a request to getting a response, and the server does not do anything and immediately responds after receiving the request. The experiment was conducted in the following two cases to observe the transmission cost caused by the introduction of OTP:

1. Request without OTP
2. Request with OTP

As can be seen from Table 5, requests with the OTP are 0.3 ms longer than requests without the OTP, so we can think that requests with the OTP will bring an additional 0.3 ms of transmission cost. Therefore, the transmission cost is negligible.

### 5.3. Comparison

To better understand the difference between MOTP and other schemes, we compare MOTP with classical schemes, i.e., Lamport’s OTP and TOTP. Besides, we compare MOTP with recent works, i.e., T/Key and SmartOTPs. We refer to [26] for the four properties of comparison, and Table 6 shows a property comparison between five schemes. + represents that the scheme supports the property, and − means that the scheme does not support it.

In Table 6, we can observe that except for TOTP, the other four schemes can resist leakage attacks against the server. Although MOTP cannot generate OTPs infinitely, like TOTP, MOTP needs to be reinitialized when all OTPs are consumed, reducing the risk of the scheme being cracked. In terms of OTPs’ validity, TOTP, T/Key, and MOTP make OTPs time sensitive to improve the security of OTPs. In terms of implementation, SmartOTPs is more challenging to implement and deploy than other schemes, because it involves four entities and the other schemes involve two entities: a client and a server.

After a simple comparison of the five schemes, we compare in detail MOTP with T/Key and SmartOTPs.

#### 5.3.1. Comparison with T/Key

To improve the performance of T/Key, Kogan et al. proposed the idea of the “checkpoint” to improve OTP generation efficiency [8]. The checkpoint is a node of the hash chain and is stored on the client to save the number of hash operations.

For example, select one checkpoint from every $2^{10}$ nodes on the hash chain of length $2^{20}$, and get a total of $2^{10}$ checkpoints. All checkpoints are stored on the client.

When the client generates an OTP, it will perform hash operations on the most recent checkpoint, thus reducing the number of hash operations. The checkpoint improves OTP generation efficiency in the storage cost, but it does not improve the worst-case OTP verification efficiency. If we want to improve the efficiency of OTP verification, we can introduce checkpoints in the server. However, this approach is not recommended. This is because storing checkpoints of many users will impose a storage burden on the server, and the leaked checkpoints will increase the probability of the hash chain being cracked.

Assume that MOTP and T/Key both can provide $2^{h_{m}}\;p$ OTPs. Table 7 shows the space-time comparison of the two schemes.

In terms of client storage, MOTP needs to store the entire Merkle tree and all the nonces, while T/Key without checkpoints only needs to store a nonce, so the cost of MOTP storage is much higher than T/Key. However, as mentioned in Section 3.3.3, MOTP provides 1,048,576 OTPs, which only requires 96KB of client storage, which is tolerable for typical mobile devices.

Besides, in terms of server storage, MOTP needs to store the root node of $2^{h_{m}-h_{s}}$ subtrees, while T/Key without checkpoints only needs to store a hash value. In the experiment, $h_{m}$ was set to 10, and $h_{s}$ was set to seven. Therefore, the server storage of MOTP is eight times greater than that of T/Key without checkpoints. Therefore, MOTP’s server storage is slightly higher than T/Key’s.

Moreover, in terms of time complexity, MOTP spreads the OTP generation and verification overhead on $2^{h_{m}}$ hash chains of length *p*, and T/Key without checkpoints depends on a hash chain of length $2^{h_{m}}\;p$. Therefore, the worst time complexity of MOTP generation and verification is much lower than that of T/Key. We remark that the OTP verification of MOTP needs $h_{s}$ hash operations to verify the proof path. In general, $h_{s}$ is much smaller than *p*, and $O(p+h_{s})$ can be regarded as O(p).

To make the worst time complexity of T/Key the same as MOTP, we introduced $2^{h_{m}}$ checkpoints in the client and the server, respectively. Then, we split the hash chain into $2^{h_{m}}$ hash chains of length *p*. The first node of the hash chain serves as the client’s checkpoint, and the tail node serves as the server’s checkpoint (does not consider leakage issues). Again, in the case of the same number of hash chains, because MOTP uses the root node of the Merkle tree to verify the OTP, the server storage cost of MOTP is much lower than that of T/Key with checkpoints. By comparison, MOTP improves OTP generation and verification performance, at the cost of little server storage and tolerable client storage.

#### 5.3.2. Comparison with SmartOTPs

SmartOTPs also uses a Merkle tree and hash chains to generate OTPs. Nevertheless, they are quite different in other ways.

1. In terms of the entities involved, SmartOTPs involves four entities: an authenticator (mobile), a client, a private key wallet, and a smart contract. MOTP only involves two entities: a client and a server.
2. In terms of OTP usability, SmartOTPs separates the construction of OTP={otp,proof} on two devices. The authenticator is used to generate otp so that the user only needs to enter the short digit otp to the client. Then, the client generates proof and sends {otp,proof} to the smart contract. On the other hand, MOTP uses QR codes to transmit the OTP generated by the client.
3. In the use of the hash chain, SmartOTPs only uses Lamport’s hash chain, while MOTP adopts the idea of T/Key and introduces a time gap to make OTP time sensitive.
4. In terms of OTP location, SmartOTPs relies on the sequence number sent by the client to the authenticator to generate the corresponding OTP. On the other hand, MOTP uses the current time of the device to generate the corresponding OTP without relying on other devices.

## 6. Conclusions and Future Work

Inspired by T/Key, we propose in this paper the Merkle tree-based OTP algorithm, called MOTP. MOTP constructs time sensitive OTPs to improve the security of the generated OTPs. Moreover, the periodic re-initialization of MOTP can reduce the probability of the root node being cracked. Compared with T/Key, MOTP can significantly improve OTP generation and verification efficiency at the cost of little server storage and tolerable client storage. Compared with the two-factor authentication scheme based on TOTP, the scheme based on MOTP can resist leakage attacks against the server. Again, security analysis and experimental results show that MOTP has adequate security and brings little delay to two-factor authentication.

As future work, we intend to design and assess the configuration parameter’s standard values of MOTP and shorten the length of the OTP while ensuring the same security level [27], as well as investigate techniques for resource protection and recovery [28,29,30].

## Figures and Tables

**Table 1:** Notation description. OTP, One-Time Password.

Notation	Description
$h_{m}$	Height of the Merkle tree (the height of the root node is 0)
$h_{s}$	Height of the subtree
*p*	Execution times of the hash function when constructing a hash chain
hash(x)	Hash *x*
$hash^{p}\left(x\right)$	Hash *x* *p* times
$t_{gen}$	Generation time of the Merkle tree
$t_{minus}$	Difference between the device’s current time and $t_{gen}$
$t_{gap}$	Time gap of the OTP
otp	Node value on the hash chain
proof	Proof path

**Table 2:** The relationship between the height of the Merkle tree, the number of OTPs, and the client storage (the size of the hash value and the nonce is 32 B).

Height	Number of OTPs	Client Storage
10	1024	96 KB
15	32,768	3 MB
20	1,048,576	96 MB

**Table 3:** MOTP parameter configuration.

Parameter	Value	Parameter	Value
$h_{m}$	10	*p*	1024
$h_{s}$	7	Hash algorithm	SHA256
$t_{gap}$ (s)	30	Size of nonce (bit)	256

**Table 4:** Initialization time cost of T/Key and MOTP (ms).

Validity Period (Year)	Mobile		PC	
	T/Key	MOTP	T/Key	MOTP
1	5403	5445	1193	1280
2	10791	10,809	2356	2409
4	21,610	21,540	4713	4812

**Table 5:** Average time for response.

	Request without OTP	Request with OTP
Time (ms)	0.7811	1.0923

**Table 6:** Scheme comparison. TOTP, Time-based One-Time Password.

Scheme	Resistance to Leakage Attacks	Infinite OTPs	OTPs’ Validity Is Limited in Time	Ease of Implementation
Lamport’s OTP	+	−	−	+
TOTP	−	+	+	+
T/Key	+	−	+	+
SmartOTPs	+	−	−	−
MOTP	+	−	+	+

**Table 7:** MOTP and T/Key space-time complexity comparison (the size of the hash value and nonce is *b*).

Comparison	MOTP	T/Key without Checkpoints	T/Key with Checkpoints
Client storage	$(3\cdot 2^{h_{m}}-1)b$	*b*	$2^{h_{m}}b$
Server storage	$2^{h_{m}-h_{s}}b$	*b*	$2^{h_{m}}b$
OTP generation time complexity	O(p), Ω(1)	$O(2^{h_{m}}p)$, Ω(1)	O(p), Ω(1)
OTP verification time complexity	$O(p+h_{s})$, Ω(1)	$O(2^{h_{m}}p)$, Ω(1)	O(p), Ω(1)
